# Drug interactions in patients with alcohol use disorder: results from a real-world study on an addiction-specific ward

**DOI:** 10.1177/20420986241311214

**Published:** 2025-01-18

**Authors:** Sebastian Schröder, Christina Massarou, Tabea Pfister, Stefan Bleich, Phileas Johannes Proskynitopoulos, Johannes Heck, Martin Schulze Westhoff, Alexander Glahn

**Affiliations:** Department of Psychiatry, Social Psychiatry and Psychotherapy, Hannover Medical School, Carl-Neuberg-Str. 1, Hannover 30625, Germany; Department of Psychiatry, Social Psychiatry and Psychotherapy, Hannover Medical School, Hannover, Germany; Department of Psychiatry, Social Psychiatry and Psychotherapy, Hannover Medical School, Hannover, Germany; Department of Psychiatry, Social Psychiatry and Psychotherapy, Hannover Medical School, Hannover, Germany; Department of Psychiatry, Social Psychiatry and Psychotherapy, Hannover Medical School, Hannover, Germany; Institute for Clinical Pharmacology, Hannover Medical School, Hannover, Germany; Department of Psychiatry, Social Psychiatry and Psychotherapy, Hannover Medical School, Hannover, Germany; Department of Psychiatry, Social Psychiatry and Psychotherapy, Hannover Medical School, Hannover, Germany

**Keywords:** alcohol, alcohol–medication interactions, alcohol use disorder, drug–drug interactions, drug safety

## Abstract

**Background:**

The majority of patients with alcohol use disorder (AUD) regularly take medication. Alcohol interacts negatively with many commonly prescribed drugs. However, little is known about the characteristics and frequency of potential alcohol–medication and drug–drug interactions in patients with AUD.

**Objectives:**

This study aimed to determine the prevalence and characteristics of drug interactions in patients with AUD during withdrawal therapy on an addiction-specific ward.

**Design:**

Retrospective cohort study.

**Methods:**

Medication charts were analyzed and screened for potential alcohol–medication and drug–drug interactions. For the screening of potential alcohol–medication interactions, the drugs.com classification was utilized and potential drug–drug interactions were identified using the mediQ electronic interaction program.

**Results:**

In our study, almost two-thirds (66.3%; 1089/1643) of all patient cases were prescribed at least one drug that could potentially interact with alcohol. Four percent of all alcohol–medication interactions were classified as severe, 91.8% as moderate, and 4.3% as mild. Drug classes commonly involved in serious interactions with alcohol were analgesics and drugs used in diabetes. A total of 811 potential drug–drug interactions were identified, of which 3.3% were classified as severe and 96.5% as moderate. Psychoanaleptics (ATC N06) and psycholeptics (ATC N05) were most frequently associated with moderate to severe interactions.

**Conclusion:**

Potential alcohol–medication and drug–drug interactions are common in hospitalized patients with AUD. Improvements in the quality of prescribing should focus on the use of psychotropic drugs.

## Plain language summary

Understanding how drugs interact in people with alcohol addiction: findings from a study conducted in a specialized treatment unit

Background: People with alcohol use disorder often take multiple medications. Mixing alcohol with certain medications can cause harmful effects. We aimed to find out how frequently potential interactions occur between alcohol and medication during withdrawal therapy, as well as how frequently potential interactions occur between different medications. What we did: We reviewed the medication charts of patients receiving care in a specialized alcohol treatment unit. Using interaction-checking tools, we assessed possible interactions between alcohol and prescribed medications, as well as between different medications. Key findings: About two-thirds of patients were prescribed at least one medication that could potentially interact with alcohol. Some of these interactions were severe, particularly with painkillers and diabetes medications. We also identified possible interactions between different medications. Psychoactive drugs (medications that affect the brain, mood, or behavior) were the most common to interact with other drugs. What this means: This study highlights that interactions between alcohol and medications, and among different medications, are common in patients with alcohol use disorder. It emphasizes the need for better prescribing practices, particularly when it comes to psychoactive drugs, to improve patient safety.

## Introduction

Alcohol use disorder (AUD) and related alcohol consumption are leading risk factors for morbidity and mortality worldwide.^1^ Life expectancy of patients with AUD is reduced by up to 28 years.^2^ Individuals diagnosed with AUD are at increased risk of developing severe somatic comorbidities, such as cardiovascular, liver, and pancreatic diseases.^3,4^ Psychiatric comorbidities like depression or personality disorders are also common.^5,6^ The prevalence of multiple diagnoses often requires drug prescriptions leading to frequent polypharmacy in patients with AUD.^7^ In turn, polypharmacy represents a major risk factor for drug–drug interactions (DDIs) or the occurrence of adverse drug reactions (ADRs), which frequently necessitate admission to emergency departments and hospitalization.^8–11^ To mitigate these risks, several DDI screening programs and databases have been developed for clinical practice, providing clinicians with tools to identify and prevent potential DDIs and ADRs.^12^ These screening tools are designed to assist in the early identification of patients at risk, in particular by assessing factors such as kidney and liver function, age, and genetic predisposition.^13^

Furthermore, during alcohol consumption, patients with AUD are at additional risk of potential alcohol–medication interactions (AMIs), which can lead to changes in alcohol metabolism.^14,15^ AMIs may result in increased sedation, risk of hypoglycemia, orthostatic hypotension, susceptibility to gastrointestinal bleeding, as well as liver damage.^14^ AMI can increase peak plasma concentrations and prolong the elimination half-life of drugs such as benzodiazepines, cannabis, opioids, and methylphenidate, leading to enhanced effects or toxicity.^16^ This may increase the risk of adverse effects, including central nervous system depression or increased therapeutic drug response.^16^

Not all AMIs are dose dependent, but there is evidence that the likelihood of AMIs increases with higher doses of alcohol consumption.^17,18^ In addition, ADRs involving alcohol appear to have more serious consequences for patients than non-alcohol-related ADRs, such as an increased likelihood of hospital admission.^19^

There are a few studies that have examined the prevalence of potential AMIs at a population level, but there is no study available to date that specifically focuses on patients with a diagnosis of AUD following withdrawal treatment.^18,20^

Given the increased vulnerability of patients with AUD for ADRs, special attention needs to be paid to the appropriate prescribing of drugs in their medical care. Therefore, this study aimed to retrospectively analyze potential AMIs and DDIs in inpatients diagnosed with AUD at an addiction-specific ward during withdrawal therapy in a university hospital in Germany over 6 years.

## Methods

### Study design and eligibility criteria

The study was conducted as a retrospective cohort study. Patients were included in the study, if (i) they were treated on the addiction-specific ward of the Department of Psychiatry, Social Psychiatry and Psychotherapy of Hannover Medical School between January 2016 and December 2021, (ii) they were diagnosed with AUD, and (iii) they or their legal representative had provided written informed consent that patient-related data be used for clinical research. There were no specific exclusion criteria. Hannover Medical School is a large university hospital and tertiary care referral center in northern Germany. The addiction-specific ward is a 12-bed facility specializing in the treatment and care of patients with substance use disorders. All patients were inpatients.

### Acquisition of demographic data

Demographic characteristics—that is, age, sex, and medical diagnoses—were obtained from patient records.

### Medication evaluation tools

Drug prescriptions were analyzed by an interdisciplinary team of experts in psychiatry and clinical pharmacology. To this end, the drugs.com classification^21^ (Drugsite Trust, Auckland, New Zealand) and the electronic drug interaction program mediQ (Psychiatrische Dienste Aargau AG, mediQ Kompetenzzentrum für Medikamentensicherheit, Windisch, Switzerland) were utilized for the evaluation of potential AMIs and potential DDIs, respectively.

Drugs.com provides information on possible interactions between different drugs and psychotropic substances including alcohol. This includes data on the severity of the interaction, possible ADRs, and precautions to take. The database contains information on 525 drugs possibly involved in AMIs. Thirty-seven of the AMIs are classified as severe, 477 as moderate, and 11 as minor.

DDI checks were performed with mediQ, an electronic drug interaction program specialized in psychopharmaceuticals. mediQ categorizes the clinical severity of DDIs as “low,” “moderate,” “severe,” or “not beneficial because of the same mechanism of action.” For our study, only moderate, severe, and non-beneficial DDIs due to the same mechanism of action were considered for statistical analysis. We classified medications according to the World Health Organization’s Anatomical Therapeutic Chemical (ATC) system.^22^ The reporting of this study conforms to the STROBE statement.^23^

### Statistics

Microsoft^®^ Excel^®^ 2019 (Redmond, WA, USA) and IBM^®^ SPSS^®^ Statistics 28 (Armonk, NY, USA) were used for statistical analysis. Descriptive statistical methods were used to summarize the data. Continuous variables are depicted as means ± standard deviations (SDs) or as medians with interquartile ranges (IQRs). For categorical variables, absolute and relative frequencies were calculated. Pearson’s chi-squared test was used for inferential statistics. Due to the exploratory nature of our study, no adjustments were made for multiple testing.

## Results

### Study population and medication

**Figure 1.**
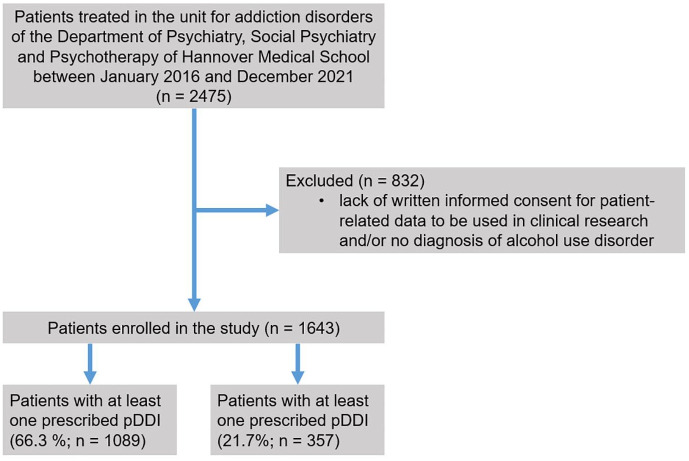
Flow of patients. pDDI, potential drug–drug interaction.

Overall, 1643 patient cases involving 830 individual patients were manually screened for potential AMIs and DDIs (Figure 1). The higher number of patient cases as compared to the number of individual patients is explained by returners. As potential AMIs and DDIs may have occurred during every hospital stay of a returning patient, each case was evaluated separately. Therefore, the following statistical analyses refer to *n* = 1643 as the denominator unless stated otherwise. The median age of the treated patient cases was 47 years (IQR 38–55 years; range 18–76 years) and more than three-quarters (75.3%; 1238/1643) of the study population were male. It should be noted that the median number of inpatient stays for the 830 individual patients was 1 (IQR 1–2 stays; range 1–31 stays). On average, each patient took 2 drugs, with an IQR of 1–4 drugs and a range of 0–23 drugs. Of all study cases, 20.2% (332/1643) were without prescribed medication. Polypharmacy, defined as the regular intake of five or more different drugs, was observed in 20.6% (339/1643) of the patient cases.

Table 1 presents an overview of the psychiatric diagnoses and somatic comorbidities within the study population. Apart from AUD, the most common psychiatric disorder was depression, which affected 34.0% (657/1643) of the patient cases. The most prevalent somatic comorbidity was arterial hypertension, which was observed in 16.6% (272/1643) of the patient cases. Moreover, 16.4% (269/1643) of the study population also had a cannabis use disorder. A total of 4889 drugs comprising 332 different individual agents were prescribed to the study population (Supplemental Table 1). The three most frequently prescribed drugs were pantoprazole (9.3%; 454/4889), levetiracetam (4.7%; 230/4889), and ramipril (4.0%; 195/4889; Supplemental Table 1).

**Table 1.** Characteristics of the study population (*n* = 1643).

Variables	*n*	%
Sex		
Female	405	24.7
Male	1238	75.3
Polypharmacy (⩾5 prescribed drugs)	339	20.6
Without prescribed medication	332	20.2
Psychiatric diagnoses^a^		
Alcohol use disorder	1643	100
Amphetamine use disorder	45	2.7
Bipolar affective disorder^b^	10	0.6
Cannabis use disorder	269	16.4
Cocaine use disorder	252	15.3
Dementia^c^	9	0.6
Delirium^d^	10	0.6
Depression^e^	657	40.0
Multiple substance dependence	56	3.4
Opioid use disorder	238	14.5
Other psychiatric disorder(s)	566	34.5
Personality disorder^f^	200	12.2
PTSD	114	6.9
Schizophrenia or schizophreniform disorder^g^	42	2.6
Sedative use disorder	153	9.3
Somatic diagnoses^a^		
Arterial hypertension	272	16.6
Atrial fibrillation	25	1.5
Chronic heart failure	30	1.8
Chronic obstructive pulmonary disease	60	3.7
Coronary heart disease	34	2.1
Epilepsy	37	2.3
Hypothyroidism	63	3.8
Other somatic disorder(s)	963	58.6
Status post stroke	27	1.6
Type-2 diabetes mellitus	103	6.3
Urinary tract infection	21	1.3

The median age (interquartile range) of the study cohort was 47 (38–55) years. The median number of inpatient stays for the 830 individual patients was 1 (1–2 stays). On average, each patient took 2 drugs (1–4 drugs).

a Patients could have more than one diagnosis;

b ICD-10 F31;

^c^ICD-10 F00, F01, F02, F03;

d ICD-10 F05;

e ICD-10 F32, F33;

f ICD-10 F60;

g ICD-10 F06.2, F20.

ICD-10, International Statistical Classification of Diseases and Related Health Problems 10th Revision; PTSD, post-traumatic stress disorder.

### Potential AMIs

With the aid of the drugs.com classification, 50.0% (2444/4889) of all prescribed drugs were identified as potentially interacting with alcohol. 4.0% (97/2444) of all AMIs were classified as severe, 91.8% (2243/2444) as moderate, and 4.3% (104/2444) as mild. In almost two-thirds (66.3%; 1089/1643) of the study cases, at least one drug potentially interacting with alcohol was prescribed. There was no statistically significant association between sex (*p* = 0.695) or between different age groups (*p* = 0.469) for the risk of prescription of a potentially major AMI.

ATC groups most affected by potential severe AMIs (*n* = 97) were analgesics (N02) and drugs used in diabetes (A10) with 64.9% (63/97) and 34.0% (33/97), respectively (Table 2).^22^ Psychoanaleptics (N06) (24.5%; 550/2243), psycholeptics (N05) (12.5%; 280/2243), and antiepileptics (N03) (12.3%; 275/2243) were most frequently involved in potential moderate AMIs (*n* = 2243; Table 2). Psycholeptics (N05) (85.6%; 89/104), antiviral agents for systemic use (J05) (9.6%; 10/104), and antibacterials for systemic use (J01) (4.8%; 5/104) were most commonly contributed to mild AMIs (*n* = 104; Table 2). The most frequently prescribed drugs associated with potential severe AMIs were buprenorphine (49.5%; 48/97), metformin (34.0%; 33/97), and morphine (8.2%; 8/97) (Supplemental Table 2). For potential moderate interactions, levetiracetam (10.3%; 230/2243), mirtazapine (6.6%; 147/2243), and sertraline (5.4%; 122/2243) were most commonly involved, while quetiapine (85.6%; 89/104), abacavir (9.6%; 10/104), and doxycycline (4.8%; 5/104) were most frequently contributed to mild AMIs (Supplemental Table 2).

**Table 2.** Prevalence of the drug categories (ATC classification) of potential alcohol–medication interactions according to the drugs.com classification (*n* = 2444).

ATC classification	*n*	%
All potential alcohol–medication interactions	2444	100
Potential severe alcohol–medication interactions	97	100
N02 Analgesics	63	64.9
A10 Drugs used in diabetes	33	34.0
A01 Stomatologics	1	1.0
Potential moderate alcohol–medication interactions	2243	100
N06 Psychoanaleptics	550	24.5
N05 Psycholeptics	280	12.5
N03 Antiepileptics	275	12.3
C03 Diuretics	236	10.5
N02 Analgesics	203	9.1
C07 Beta-blocking agents	175	7.8
C10 Lipid modifying agents	122	5.4
C08 Calcium channel blockers	119	5.3
B01 Antithrombotic agents	96	4.3
A10 Drugs used in diabetes	76	3.4
N07 Other nervous system drugs	60	2.7
A12 Mineral supplements	9	0.4
N04 Anti-Parkinson drugs	7	0.3
A13 Tonics	7	0.3
A11 Vitamins	5	0.2
G04 Urologicals	5	0.2
R06 Antihistamines for systemic use	4	0.2
C09 Agents acting on the renin–angiotensin system	4	0.2
D11 Other dermatological preparations	3	0.1
C02 Antihypertensives	2	0.1
L01 Antineoplastic agents	2	0.1
A03 Drugs for functional gastrointestinal disorders	1	0.0
M01 Anti-inflammatory and antirheumatic products	1	0.0
M03 Muscle relaxants	1	0.0
Potential mild alcohol–medication interactions	104	100
N05 Psycholeptics	89	85.6
J05 Antiviral agents for systemic use	10	9.6
J01 Antibacterials for systemic use	5	4.8

ATC, Anatomical Therapeutic Chemical.

### Potential DDIs

In total, 811 potential DDIs could be detected. Of these, 3.3% (27/811) were classified as severe, 96.5% (783/811) as moderate, and one was classified as not beneficial because of the same mechanism of action. 21.7% (357/1643) of the study population were affected by potential DDIs. More than two-thirds of the DDIs were classified as pharmacodynamic (70.7%; 573/811), 6.8% (55/811) as pharmacokinetic, and 22.4% (182/811) as mixed (i.e., involving both pharmacodynamic and pharmacokinetic mechanisms). There was a statistically significant association between sex (*p* = 0.006) for the risk of prescription of a potentially severe DDI with female patients at increased risk. With regard to age groups, there was no statistically significant correlation (*p* = 0.145) for the risk of prescription of a potentially serious DDI.

Regarding medication groups according to the ATC classification, psychoanaleptics (N06) (66.7%; 18/27), diuretics (C03) (29.6%; 8/27), mineral supplements (A12) (29.6%; 8/27), and psycholeptics (N05) (29.6%; 8/27) were most commonly involved in potential severe DDIs (Table 3). Furthermore, psycholeptics (N05) (43.6%; 341/783), psychoanaleptics (N06) (40.2%; 315/783), and analgesics (N02) (21.8%; 170/783) were the three medication classes most frequently involved in potential moderate DDIs (Table 3). The three most commonly prescribed drugs with potential for severe DDIs were potassium (29.6%; 8/27), spironolactone (29.6%; 8/27), and mirtazapine (22.2%; 6/27) (Supplemental Table 3). Most commonly prescribed drugs with potential for moderate DDIs were pipamperone (15.6%; 122/783), acetylsalicylic acid (10.0%; 78/783), as well as metamizole and ramipril (each 8.8%; 69/783) (Supplemental Table 3). The non-beneficial interaction pair due to the same mechanism of action was torasemide plus furosemide (1/811) (Supplemental Table 2).

**Table 3.** Prevalence of the drug categories (ATC classification) of potential drug–drug interactions according to mediQ (*n* = 1622).

Drug	*n*	%
All drugs involved into potential drug–drug interactions	1622	100
Drugs involved in potential severe drug–drug interactions	54	100
N06 Psychoanaleptics	18	33.3
A12 Mineral supplements	8	14.8
C03 Diuretics	8	14.8
N05 Psycholeptics	8	14.8
D11 Other dermatological preparations	4	7.4
L04 Immunosuppressants	4	7.4
N03 Antiepileptics	1	1.9
C01 Cardiac therapy	1	1.9
N02 Analgesics	1	1.9
H03 Thyroid therapy	1	1.9
Potential moderate drug–drug interactions	1566	100
N05 Psycholeptics	341	21.8
N06 Psychoanaleptics	315	20.1
N02 Analgesics	170	10.9
C03 Diuretics	138	8.8
C09 Agents acting on the renin–angiotensin system	110	7.0
B01 Antithrombotic agents	109	7.0
C10 Lipid modifying agents	64	4.1
N03 Antiepileptics	46	2.9
C08 Calcium channel blockers	39	2.5
R03 Drugs for obstructive airway diseases	39	2.5
C01 Cardiac therapy	26	1.7
C07 Beta-blocking agents	23	1.5
M04 Antigout preparations	17	1.1
C02 Antihypertensives	17	1.1
J05 Antiviral agents for systemic use	17	1.1
A10 Drugs used in diabetes	17	1.1
J01 Antibacterials for systemic use	12	0.8
A12 Mineral supplements	12	0.8
B03 Antianemic preparations	11	0.7
L04 Immunosuppressants	9	0.6
A02 Drugs for acid-related disorders	8	0.5
H03 Thyroid therapy	7	0.4
C05 Vasoprotectives	7	0.4
N07 Other nervous system drugs	3	0.2
M03 Muscle relaxants	3	0.2
R06 Antihistamines for systemic use	2	0.1
D11 Other dermatological preparations	2	0.1
N04 Anti-Parkinson drugs	1	0.1
A11 Vitamins	1	0.1
Not beneficial because the same mechanism of action	2	100
C03 diuretics	2	100.0

ATC, Anatomical Therapeutic Chemical.

## Discussion

In this study, we examined the prevalence and characteristics of potential AMIs and DDIs in patients with AUD during withdrawal treatment over a 6-year period. Our key findings show that 50.0% of all prescribed medications potentially interacted with alcohol, with 4.0% classified as severe AMIs, mainly involving analgesics and drugs used in diabetes. In addition, potential DDIs were identified in 21.7% of cases, with severe interactions occurring in 3.3% of cases, and women being at significantly higher risk of severe DDIs than men. The most common drug classes involved in potentially severe DDIs were psychoanalgesics, diuretics, and mineral supplements. Notably, this is the first study to use both the drugs.com classification for AMIs and the mediQ tool for DDIs specifically in AUD patients.

Our study population differed from previous studies in terms of age, gender, and comorbidity profiles and the applied screening tools for potential drug interactions.^18,20^ In our study, the mean age of the study population was approximately 47 years, and the most common psychiatric diagnosis apart from AUD was depression. Several prior investigations have explored the characteristics of drug interactions in the context of alcohol consumption on a general population level.^18,20^ These studies consistently showed that a significant proportion of prescribed drugs had the potential to interact with alcohol.^18,20^ The reported prevalence of AMIs was heterogeneous, ranging from 13% to 42%.^18,20^ Such discrepancies might be ascribed to different study designs and settings. In previous investigations, the most commonly prescribed drugs involved in potential AMIs were benzodiazepines and antipsychotics and drugs for the treatment of cardiovascular diseases.^18,20^

Regarding geriatric populations, numerous studies have examined the characteristics of potential AMIs.^24–27^ A systematic review conducted by Holton et al. found that between 21% and 35% of older adults might be affected by potential AMIs.^28^ In a cohort of geriatric inpatients with AUD, Schröder et al. identified potentially significant interactions between alcohol and prescribed drugs in over 80%.^29^

The results of the present study also suggest that a significant proportion of drugs prescribed to patients with AUD should be assessed critically due to their potential to interact with alcohol. However, it is important to note that the drugs.com classification was not originally developed for use in patients with AUD, but rather for the assessment of potential AMIs in general.

The ATC groups mostly involved in potential severe AMIs were analgesics (N02) and drugs used in diabetes (A10), while buprenorphine, metformin, and morphine represented the most common drugs involved in severe AMIs. Prescription of opioids is a common phenomenon in patients with AUD and often refers to substitution in the context of coexisting opioid use disorders or analgetic therapy.^30^ Opioid use disorder also affected 14.5% (238/1643) of our collective. The findings of Jobski et al. also indicate frequent pharmacokinetic interactions between orally administered opioids and alcohol consumption, resulting in a significant increase in the incidence of ADRs, highlighting the clinical relevance.^31^ A rational evaluation of medications in addiction psychiatry requires careful benefit–risk analyses and consideration of alternative pharmacological options. This is particularly challenging in patients with concurrent AUD and opioid dependence under substitution therapy, as opioids—especially buprenorphine—can have dangerous interactions with alcohol. In line with the medical principle of “do no harm” (primum non nocere), as outlined in the Hippocratic Oath, patient safety must always be the priority. However, it is equally important to assess the potential risks of withholding treatment. In this context, clinicians must carefully weigh the risks of severe interactions between alcohol and buprenorphine while also considering the broader impact of substance use disorders on quality of life, the prognosis of comorbid somatic conditions, and the increased risk of suicidality.

In addition, prescribing biguanides such as metformin to patients with AUD significantly increases the risk of potentially fatal lactic acidosis; therefore, it is contraindicated.^32,33^

Sodium glucose linked transporter 2 (SGLT-2) inhibitors should be considered as a therapeutic alternative, but they also carry an increased risk of diabetic ketoacidosis when used in combination with alcohol.^34^ Promising early studies suggest that prescribing Glucagon-Like Peptide 1 (GLP-1) agonists to patients with type-2 diabetes mellitus and AUD may lead to a reduction in alcohol consumption.^35,36^

With regard to potential moderate interactions between alcohol and drugs, psychoanaleptics (N06), psycholeptics (N05), and antiepileptics (N03) were the most frequent and were mainly related to the drugs levetiracetam, mirtazapine, and sertraline. The high proportion of psychoanaleptics and psycholeptics may be explained by the high proportion of psychiatric comorbidity in our population, which is well known in patients with AUD.^37^ Although patients with AUD are at increased risk of withdrawal seizures, the long-term prescription of antiepileptic drugs without a diagnosis of epilepsy must always be critically questioned due to the frequent lack of approval.^38^ Levetiracetam in particular is often prescribed in such patients.^39^ In particular, levetiracetam is not a suitable long-term treatment for AUD, partly because there is evidence that it increases the amount of alcohol consumed.^40^ The clinical use of antiepileptic drugs specifically for the prophylaxis of alcohol withdrawal seizures cannot be recommended in the absence of an underlying diagnosis of epilepsy.^41^ With regard to mirtazapine, alcohol appears to potentiate the adverse effects of sedative antidepressants at the pharmacodynamic level, although the mechanisms of these interactions are poorly understood.^42^ Sertraline is commonly prescribed for many psychiatric conditions and—as all selective serotonin reuptake inhibitors—exerts a negative effect on platelet aggregation, which is also impaired in many AUD patients, increasing the risk of bleeding.^43,44^ It is important to note that there is some evidence that selective serotonin reuptake inhibitors may not only be ineffective in people with AUD but may actually worsen AUD.^45^ Overall, the limited number of randomized controlled trials (RCTs) suggests that no recommendation can be made for the use of antidepressant medication in the treatment of depressive symptoms associated with AUD.^46^

In terms of potential mild interactions, psycholeptics (N05), antiviral agents for systemic use (J05), and antibacterials for systemic use (J01) were mostly involved, especially quetiapine, abacavir, and doxycycline. In particular, the sedative effect of quetiapine may lead to synergistic effects when taken together with alcohol.^47^ Abacavir is metabolized by alcohol dehydrogenase, resulting in reduced formation and elimination of metabolites when used in patients with impaired liver function and is therefore contraindicated in moderate or severe hepatic impairment, which occurs in many patients with AUD.^48,49^ Doxycycline is known to be less effective in chronic alcohol use.^50^

The four medication groups most frequently involved in potential severe DDIs were psychoanaleptics (N06), diuretics (C03), mineral supplements (A12), and psycholeptics (N05). On the individual drug level, potassium, spironolactone, and mirtazapine were most frequently implicated in potential severe DDIs. The concomitant use of inhibitors of the renin–angiotensin–aldosterone system and potassium can lead to potentially fatal hyperkalemia, especially in patients with impaired renal function, and should therefore only be used under close laboratory monitoring.^51^ Mirtazapine has many known drug interactions, also with easily available over-the-counter medicines.^52^

The three drug groups most frequently involved in potential moderate DDIs were psycholeptics (N05), psychoanaleptics (N06), and analgesics (N02). Individual drugs most commonly involved in potential moderate DDIs were pipamperone, acetylsalicylic acid, as well as metamizole and ramipril with a shared third rank. Pipamperone is often used in combination with other psychotropic drugs specifically due to its sedative potential, but such combinations appear to significantly increase the risk of QTc interval prolongation with the potentially fatal complication of torsades de pointes arrhythmias.^53 54^ For the combination of acetylsalicyclic acid and metamizole, there is evidence of impaired platelet inhibition.^55^ Particularly in patients with impaired liver function, which many patients with AUD suffer from, special care and diligent risk–benefit assessments are required for appropriate pain management.^56^ In a study by Shehab et al., renin–angiotensin–aldosterone system inhibitors were among the drugs most frequently associated with US emergency department visits due to adverse drug events, leading to hospitalization in up to 25% of cases, highlighting the clinical relevance of this potential interaction.^57^

In conclusion, our study indicates that in the field of addiction psychiatry, a significant number of patients with AUD receive medications with the potential to interact with alcohol. Furthermore, the number of prescriptions with potential AMIs represents a significant proportion of total prescriptions. The use of interaction checks to assess the appropriateness of medications for the addiction setting appears to be beneficial in improving medication safety for this patient cohort. Therefore, a comprehensive assessment of prescribed medications in patients with AUD requires a thorough analysis of benefits and risks, as well as careful consideration of alternative pharmacological options. However, a limitation of our study is that the classifications we applied are not explicitly tailored to psychiatric contexts. In particular, the drugs.com classification does not provide pharmacological alternatives. The selection of medications analyzed in our study might also be a point for criticism. These drugs were taken from the list of drugs prescribed to patients after their inpatient treatment. The therapeutic goal is to achieve alcohol abstinence after qualified withdrawal therapy. In theory, AMIs should not occur after discharge from the hospital. Unfortunately, relapse to alcohol use after discharge is not uncommon.^58^ Consequently, AMIs are a significant problem in clinical practice that healthcare providers need to be aware of and familiar with. Screening and brief interventions should be provided for AUDs and the use of medications that interact with alcohol.^59^ Harm reduction measures should be considered for individuals who are concurrently using alcohol, including consideration of alternative, lower-risk medications.

Although many studies have investigated AMIs, there is currently no consensus on the definition of alcohol-interacting medications. There is an urgent need to establish a clear and validated list of potential alcohol-interacting medications for patients with AUD through prospective research.

Pharmacies and clinics have the potential to both raise awareness of the risk of AMIs and reduce alcohol consumption.^60,61^

The limitations of our study include its monocentric design and the exclusive setting within a specialized unit of a university hospital. Consequently, the generalizability of our findings to other healthcare settings may be limited. In addition, the retrospective nature of our study prevents us from assessing whether the identified potential AMIs or DDIs resulted in adverse outcomes in our study population. To address these limitations, future research should adopt a prospective design to comprehensively analyze the true risk of adverse outcomes associated with potential AMIs and DDIs in patients with AUD. Such prospective studies will allow healthcare professionals to effectively stratify individuals with AUD based on their risk profiles at the time of prescription. In addition, RCTs should be conducted to prospectively investigate whether mitigation of potential AMIs and DDIs can actually reduce the incidence of adverse effects in patients with AUD.

## Supplemental Material

sj-docx-1-taw-10.1177_20420986241311214 – Supplemental material for Drug interactions in patients with alcohol use disorder: results from a real-world study on an addiction-specific ward Supplemental material, sj-docx-1-taw-10.1177_20420986241311214 for Drug interactions in patients with alcohol use disorder: results from a real-world study on an addiction-specific ward by Sebastian Schröder, Christina Massarou, Tabea Pfister, Stefan Bleich, Phileas Johannes Proskynitopoulos, Johannes Heck, Martin Schulze Westhoff and Alexander Glahn in Therapeutic Advances in Drug Safety

## Appendix

### Abbreviations

ADR Adverse drug reaction

AMI Alcohol–medication interaction

AUD Alcohol use disorder

DDI Drug–drug interaction

ICD-10 International Statistical Classification of Diseases and Related Health Problems 10th Revision

IQR Interquartile range

## Declarations

**Ethics approval and consent to participate:** The study was approved by the Ethics Committee of Hannover Medical School (No. 10764_BO_K_2023) and adhered to the Declaration of Helsinki and its later amendments. Written informed consent was obtained from all patients or their legal representatives before their inclusion in the study.

**Consent for publication:** Patients were only included if they or their legal representative gave written informed consent for patient-related data to be published for research.

**Availability of data and materials:** The data that support the findings of this study are available upon reasonable request from the corresponding author.
