# Supplementary material for: Drug interactions in patients with alcohol use disorder: results from a real-world study on an addiction-specific ward
Source: Ther Adv Drug Saf. 2025 Jan 18;16:20420986241311214. doi: 10.1177/20420986241311214 (PMC11742168; doi:10.1177/20420986241311214)
Supplement: sj-docx-1-taw-10.1177_20420986241311214 – Supplemental material for Drug interactions in patients with alcohol use disorder: results from a real-world study on an addiction-specific ward [file sj-docx-1-taw-10.1177_20420986241311214.docx]

**SUPPLEMENTARY TABLE 1 All prescribed medications** (n = 4889) **in the study population**

| **Drug** | **n** | **%** |
| --- | --- | --- |
| **All prescribed medications** | **4889** | **100** |
| Pantoprazole | 454 | 9.3 |
| Levetiracetam | 230 | 4.7 |
| Ramipril | 195 | 4.0 |
| Pipamperone | 149 | 3.0 |
| Mirtazapine | 147 | 3.0 |
| Sertraline | 122 | 2.5 |
| Amlodipine | 117 | 2.4 |
| Bisoprolol | 103 | 2.1 |
| Torasemide | 94 | 1.9 |
| Acetylsalicylic acid | 92 | 1.9 |
| Oxazepam | 89 | 1.8 |
| Quetiapine | 89 | 1.8 |
| L-thyroxine | 80 | 1.6 |
| Levomethadone | 78 | 1.6 |
| Venlafaxine | 76 | 1.6 |
| Metamizole | 75 | 1.5 |
| Thiamine | 74 | 1.5 |
| Vitamin B | 70 | 1.4 |
| Spironolactone | 64 | 1.3 |
| Atorvastatin | 62 | 1.3 |
| Pregabalin | 61 | 1.2 |
| Naltrexone | 60 | 1.2 |
| Simvastatin | 57 | 1.2 |
| Hydrochlorothiazide | 51 | 1.0 |
| Metoprolol | 51 | 1.0 |
| Magnesium | 50 | 1.0 |
| Buprenorphine | 48 | 1.0 |
| Formoterol | 48 | 1.0 |
| Candesartan | 46 | 0.9 |
| Vitamin D | 44 | 0.9 |
| Risperidone | 41 | 0.8 |
| Folic acid | 38 | 0.8 |
| Agomelatine | 36 | 0.7 |
| Doxepin | 36 | 0.7 |
| Salbutamol | 36 | 0.7 |
| Human insulin | 35 | 0.7 |
| Aripiprazole | 33 | 0.7 |
| Metformin | 33 | 0.7 |
| Lactulose | 32 | 0.7 |
| Insulin glargine | 31 | 0.6 |
| Citalopram | 30 | 0.6 |
| Allopurinol | 28 | 0.6 |
| Bupropion | 28 | 0.6 |
| Ibuprofen | 27 | 0.6 |
| Macrogol | 27 | 0.6 |
| Apixaban | 26 | 0.5 |
| Fluoxetine | 26 | 0.5 |
| Potassium | 26 | 0.5 |
| Cetirizine | 24 | 0.5 |
| Carbamazepine | 23 | 0.5 |
| Lorazepam | 23 | 0.5 |
| Furosemide | 22 | 0.4 |
| Valerian | 20 | 0.4 |
| Beclometasone | 20 | 0.4 |
| Chlorprothixene | 20 | 0.4 |
| Clonidine | 19 | 0.4 |
| Ipratropium | 19 | 0.4 |
| Olanzapine | 19 | 0.4 |
| Sitagliptin | 19 | 0.4 |
| Dolutegravir | 18 | 0.4 |
| Fenoterol | 18 | 0.4 |
| Ciclopirox | 17 | 0.3 |
| Tiotropium | 16 | 0.3 |
| Duloxetine | 15 | 0.3 |
| Insulin aspart | 15 | 0.3 |
| Opipramol | 15 | 0.3 |
| Pancreatin | 15 | 0.3 |
| Tenofovir | 15 | 0.3 |
| Budesonide | 14 | 0.3 |
| Diazepam | 14 | 0.3 |
| Omeprazole | 14 | 0.3 |
| Acamprosate | 13 | 0.3 |
| Fluticasone | 13 | 0.3 |
| Tilidine | 13 | 0.3 |
| Trimipramine | 13 | 0.3 |
| Valproic acid | 13 | 0.3 |
| Amoxicillin | 12 | 0.2 |
| Dapagliflozin | 12 | 0.2 |
| Iron II complex | 12 | 0.2 |
| Escitalopram | 12 | 0.2 |
| Esomeprazole | 12 | 0.2 |
| Gabapentin | 12 | 0.2 |
| Glycopyrronium | 12 | 0.2 |
| Milnacipran | 12 | 0.2 |
| Promethazine | 12 | 0.2 |
| Rivaroxaban | 12 | 0.2 |
| Carvedilol | 11 | 0.2 |
| Enalapril | 11 | 0.2 |
| Abacavir | 10 | 0.2 |
| Emtricitabine | 10 | 0.2 |
| Haloperidol | 10 | 0.2 |
| Lamivudine | 10 | 0.2 |
| Methylphenidate | 10 | 0.2 |
| Valsartan | 10 | 0.2 |
| Amisulpride | 9 | 0.2 |
| Ezetimibe | 9 | 0.2 |
| Insulin detemir | 9 | 0.2 |
| Melperone | 9 | 0.2 |
| Salmeterol | 9 | 0.2 |
| Valdoxan | 9 | 0.2 |
| Morphine | 9 | 0.2 |
| Sodium chloride | 8 | 0.2 |
| Oxycodone | 8 | 0.2 |
| Paroxetine | 8 | 0.2 |
| Zolpidem | 8 | 0.2 |
| Ciprofloxacin | 8 | 0.2 |
| Clindamycin | 7 | 0.1 |
| Enoxaparin | 7 | 0.1 |
| Fentanyl | 7 | 0.1 |
| Indacaterol | 7 | 0.1 |
| Insulin lispro | 7 | 0.1 |
| Rifaximin | 7 | 0.1 |
| Sucralfate | 7 | 0.1 |
| Tianeptine | 7 | 0.1 |
| Cotrimoxazole | 7 | 0.1 |
| Dabigatran | 6 | 0.1 |
| Empagliflozin | 6 | 0.1 |
| Iodide | 6 | 0.1 |
| Lithium | 6 | 0.1 |
| Mometasone | 6 | 0.1 |
| Sodium bicarbonate | 6 | 0.1 |
| Nebivolol | 6 | 0.1 |
| Penicillin | 6 | 0.1 |
| Tinzaparin sodium | 6 | 0.1 |
| Trazodone | 6 | 0.1 |
| Aclidinium bromide | 6 | 0.1 |
| Amitriptyline | 5 | 0.1 |
| Calcium | 5 | 0.1 |
| Clavulanic acid | 5 | 0.1 |
| Digitoxin | 5 | 0.1 |
| Doxycycline | 5 | 0.1 |
| Fresubin® | 5 | 0.1 |
| Urea | 5 | 0.1 |
| Insulin degludec | 5 | 0.1 |
| Caffeine | 5 | 0.1 |
| Octenisept® | 5 | 0.1 |
| Prednisolone | 5 | 0.1 |
| Simeticone | 5 | 0.1 |
| Tamsulosin | 5 | 0.1 |
| Lacosamide | 5 | 0.1 |
| Dalteparin | 5 | 0.1 |
| Dulaglutide | 4 | 0.1 |
| Eplerenone | 4 | 0.1 |
| Erythropoietin | 4 | 0.1 |
| Febuxostat | 4 | 0.1 |
| Fluconazole | 4 | 0.1 |
| Flupentixol | 4 | 0.1 |
| Hydromorphone | 4 | 0.1 |
| Losartan | 4 | 0.1 |
| Movicol® | 4 | 0.1 |
| Noscapine | 4 | 0.1 |
| Olmesartan | 4 | 0.1 |
| Phenprocoumon | 4 | 0.1 |
| Propranolol | 4 | 0.1 |
| Telmisartan | 4 | 0.1 |
| Vitamin C | 4 | 0.1 |
| Zinc orotate | 4 | 0.1 |
| Zuclopenthixol | 4 | 0.1 |
| Biotin | 4 | 0.1 |
| Biperiden | 3 | 0.1 |
| Clomipramine | 3 | 0.1 |
| Clopidogrel | 3 | 0.1 |
| Cobicistat | 3 | 0.1 |
| Darunavir | 3 | 0.1 |
| Dequalinium chloride | 3 | 0.1 |
| Donepezil | 3 | 0.1 |
| Edoxaban | 3 | 0.1 |
| Glibenclamide | 3 | 0.1 |
| Hydrocortisone | 3 | 0.1 |
| Hypromellose | 3 | 0.1 |
| Lamotrigine | 3 | 0.1 |
| Levomepromazine | 3 | 0.1 |
| Magaldrate | 3 | 0.1 |
| Mycophenolate mofetil | 3 | 0.1 |
| Nicotinamide | 3 | 0.1 |
| Olodaterol | 3 | 0.1 |
| Ornithine aspartate | 3 | 0.1 |
| Paracetamol | 3 | 0.1 |
| Polidocanol | 3 | 0.1 |
| Prasugrel | 3 | 0.1 |
| Sacubitril | 3 | 0.1 |
| Trehalose | 3 | 0.1 |
| Triclosan | 3 | 0.1 |
| Urea | 3 | 0.1 |
| Ursodeoxycholic acid | 3 | 0.1 |
| Vilanterol | 3 | 0.1 |
| Vitamin B12 | 3 | 0.1 |
| Xipamide | 3 | 0.1 |
| Zopiclone | 3 | 0.1 |
| Acetylcysteine | 3 | 0.1 |
| Algeldrate | 2 | 0.0 |
| Ampicillin | 2 | 0.0 |
| Atomoxetine | 2 | 0.0 |
| Carbomer eye drops | 2 | 0.0 |
| Chlorthalidone | 2 | 0.0 |
| Clarithromycin | 2 | 0.0 |
| Corticosteroid cream | 2 | 0.0 |
| Digoxin | 2 | 0.0 |
| Doxazosin | 2 | 0.0 |
| Ertugliflozin | 2 | 0.0 |
| Estradiol | 2 | 0.0 |
| Etoricoxib | 2 | 0.0 |
| Flumetasone | 2 | 0.0 |
| Fluvastatin | 2 | 0.0 |
| Fluvaxamine | 2 | 0.0 |
| Irbesartan | 2 | 0.0 |
| St. John's wort | 2 | 0.0 |
| Lercanidipine | 2 | 0.0 |
| Levocetirizine | 2 | 0.0 |
| Levodopa | 2 | 0.0 |
| Levofloxacin | 2 | 0.0 |
| Loratadine | 2 | 0.0 |
| Mesalazine | 2 | 0.0 |
| Methotrexate | 2 | 0.0 |
| Methylprednisolone | 2 | 0.0 |
| Montelukast | 2 | 0.0 |
| Multivitamin | 2 | 0.0 |
| Nitroglycerin | 2 | 0.0 |
| Norfloxacin | 2 | 0.0 |
| Nystatin | 2 | 0.0 |
| Ofloxacin | 2 | 0.0 |
| Omega-3 acid ethyl ester | 2 | 0.0 |
| Phytomenadione | 2 | 0.0 |
| Povidone | 2 | 0.0 |
| Progesterone | 2 | 0.0 |
| Ranitidine | 2 | 0.0 |
| Ranolazine | 2 | 0.0 |
| Rotigotine | 2 | 0.0 |
| Solifenacin | 2 | 0.0 |
| Sulbactam | 2 | 0.0 |
| Tacrolimus | 2 | 0.0 |
| Topiramate | 2 | 0.0 |
| Umeclidinium bromide | 2 | 0.0 |
| Unacid | 2 | 0.0 |
| Verapamil | 2 | 0.0 |
| Vitamin B2 | 2 | 0.0 |
| Vitamin B6 | 2 | 0.0 |
| Adalimumab | 1 | 0.0 |
| Aliskiren | 1 | 0.0 |
| Ambroxol | 1 | 0.0 |
| Benserazide | 1 | 0.0 |
| Benzbromarone | 1 | 0.0 |
| Dexpanthenol | 1 | 0.0 |
| Beta-acetyldigoxin | 1 | 0.0 |
| Brimonidine | 1 | 0.0 |
| Brinzolamide | 1 | 0.0 |
| Calcipotriol ointment | 1 | 0.0 |
| Calcium salt | 1 | 0.0 |
| Cannabidiol | 1 | 0.0 |
| Cefpodoxime | 1 | 0.0 |
| Ceftriaxone | 1 | 0.0 |
| Cefuroxime | 1 | 0.0 |
| Certoparin sodium | 1 | 0.0 |
| Quinine sulfate | 1 | 0.0 |
| Vitamine B5 | 1 | 0.0 |
| Cilostazol | 1 | 0.0 |
| Cimicifuga rhizome dry extract | 1 | 0.0 |
| Clemastine | 1 | 0.0 |
| Clioquinol | 1 | 0.0 |
| Clozapine | 1 | 0.0 |
| Crataegutt® | 1 | 0.0 |
| Desogestrel | 1 | 0.0 |
| Dexamethasone | 1 | 0.0 |
| Diclofenac | 1 | 0.0 |
| Dimenhydrinate | 1 | 0.0 |
| Dimethyl fumarate | 1 | 0.0 |
| Disodium hydrogen phosphate | 1 | 0.0 |
| Drospirenone | 1 | 0.0 |
| Econazole nitrate | 1 | 0.0 |
| Etericoxib | 1 | 0.0 |
| Femoston® conti | 1 | 0.0 |
| Ferric carboxymaltose | 1 | 0.0 |
| Flunitrazepam | 1 | 0.0 |
| Flupirtine | 1 | 0.0 |
| Fondaparinux | 1 | 0.0 |
| Antimycotic ointment | 1 | 0.0 |
| Progestogen | 1 | 0.0 |
| Ginkgo biloba | 1 | 0.0 |
| Glimepiride | 1 | 0.0 |
| Heparin | 1 | 0.0 |
| Hydroxycarbamide | 1 | 0.0 |
| Hydroxyzine | 1 | 0.0 |
| Ichtholan® | 1 | 0.0 |
| Indapamide | 1 | 0.0 |
| Isoleucine | 1 | 0.0 |
| Isosorbide dinitrate | 1 | 0.0 |
| Ivabradine | 1 | 0.0 |
| Ivermectin | 1 | 0.0 |
| Lenvatinib | 1 | 0.0 |
| Leucine | 1 | 0.0 |
| Lidocaine | 1 | 0.0 |
| Liraglutide | 1 | 0.0 |
| Lisdexamfetamine | 1 | 0.0 |
| Lisinopril | 1 | 0.0 |
| L-ornithine-L-aspartate | 1 | 0.0 |
| Milk thistle fruit dry extract | 1 | 0.0 |
| Medrogestone | 1 | 0.0 |
| Seawater nasal spray | 1 | 0.0 |
| Metoclopramide | 1 | 0.0 |
| Metronidazole | 1 | 0.0 |
| Lactic acid suppositories | 1 | 0.0 |
| Moclobemide | 1 | 0.0 |
| Agni casti fructus | 1 | 0.0 |
| Naftidrofuryl | 1 | 0.0 |
| Naproxen | 1 | 0.0 |
| Sodium picosulfate | 1 | 0.0 |
| Norethisterone | 1 | 0.0 |
| Ursodeoxycholic acid | 1 | 0.0 |
| Estrogens | 1 | 0.0 |
| Panthenol | 1 | 0.0 |
| Pimecrolimus cream | 1 | 0.0 |
| Piperacillin | 1 | 0.0 |
| Piracetam | 1 | 0.0 |
| Polyhexanide | 1 | 0.0 |
| Polysulfonic acid | 1 | 0.0 |
| Raltegravir | 1 | 0.0 |
| Rilpivirine | 1 | 0.0 |
| Ringer's solution | 1 | 0.0 |
| Rosuvastatin | 1 | 0.0 |
| Rupatadine | 1 | 0.0 |
| Selenium | 1 | 0.0 |
| Sofosbuvir | 1 | 0.0 |
| Coal tar solution | 1 | 0.0 |
| Tazobactam | 1 | 0.0 |
| Thiamazole | 1 | 0.0 |
| Tizanidine | 1 | 0.0 |
| Triamcinolone acetonide | 1 | 0.0 |
| Trometamol | 1 | 0.0 |
| Valine | 1 | 0.0 |
| Velpatasvir | 1 | 0.0 |
| Vitamin K1 | 1 | 0.0 |
| Xylometazoline | 1 | 0.0 |

**SUPPLEMENTARY TABLE 2** Prescribed drugs with potential interactions with alcohol according to the drugs.com classification (n = 2444)

| **Drug** | **n** | **%** |
| --- | --- | --- |
| **All potential alcohol-medication interactions** | **2444** | **100** |
| **Potential severe alcohol-medication interactions** | **97** | **100** |
| Buprenorphine | 48 | 49.5 |
| Metformin | 33 | 34.0 |
| Morphine | 8 | 8.2 |
| Hydromorphone | 4 | 4.1 |
| Paracetamol | 3 | 3.1 |
| Metronidazole | 1 | 1.0 |
| **Potential moderate alcohol-medication interactions** | **2243** | **100** |
| Levetiracetam | 230 | 10.3 |
| Mirtazapine | 147 | 6.6 |
| Sertraline | 122 | 5.4 |
| Amlodipine | 117 | 5.2 |
| Bisoprolol | 103 | 4.6 |
| Torasemide | 94 | 4.2 |
| Acetylsalicylic acid | 92 | 4.1 |
| Oxazepam | 89 | 4.0 |
| Levomethadone | 78 | 3.5 |
| Venlafaxine | 76 | 3.4 |
| Spironolactone | 64 | 2.9 |
| Atorvastatin | 62 | 2.8 |
| Pregabalin | 61 | 2.7 |
| Naltrexone | 60 | 2.7 |
| Simvastatin | 57 | 2.5 |
| Hydrochlorothiazide | 51 | 2.3 |
| Metoprolol | 51 | 2.3 |
| Risperidone | 41 | 1.8 |
| Doxepin | 36 | 1.6 |
| Human insulin | 35 | 1.6 |
| Aripiprazole | 33 | 1.5 |
| Citalopram | 30 | 1.3 |
| Bupropion | 28 | 1.2 |
| Ibuprofen | 27 | 1.2 |
| Fluoxetine | 26 | 1.2 |
| Carbamazepine | 23 | 1.0 |
| Lorazepam | 23 | 1.0 |
| Furosemide | 22 | 1.0 |
| Valerian | 20 | 0.9 |
| Olanzapine | 19 | 0.8 |
| Sitagliptin | 19 | 0.8 |
| Duloxetine | 15 | 0.7 |
| Insulin aspart | 15 | 0.7 |
| Diazepam | 14 | 0.6 |
| Trimipramine | 13 | 0.6 |
| Valproic acid | 13 | 0.6 |
| Escitalopram | 12 | 0.5 |
| Gabapentin | 12 | 0.5 |
| Milnacipran | 12 | 0.5 |
| Promethazine | 12 | 0.5 |
| Carvedilol | 11 | 0.5 |
| Haloperidol | 10 | 0.4 |
| Methylphenidate | 10 | 0.4 |
| Insulin detemir | 9 | 0.4 |
| Tramadol | 9 | 0.4 |
| Oxycodone | 8 | 0.4 |
| Paroxetine | 8 | 0.4 |
| Zolpidem | 8 | 0.4 |
| Fentanyl | 7 | 0.3 |
| Insulin lispro | 7 | 0.3 |
| Lithium | 6 | 0.3 |
| Nebivolol | 6 | 0.3 |
| Trazodone | 6 | 0.3 |
| Amitriptyline | 5 | 0.2 |
| Insulin degludec | 5 | 0.2 |
| Tamsulosin | 5 | 0.2 |
| Dulaglutide | 4 | 0.2 |
| Eplerenone | 4 | 0.2 |
| Lacosamide | 4 | 0.2 |
| Phenprocoumon | 4 | 0.2 |
| Propranolol | 4 | 0.2 |
| Telmisartan | 4 | 0.2 |
| Biperiden | 3 | 0.1 |
| Clomipramine | 3 | 0.1 |
| Lamotrigine | 3 | 0.1 |
| Levomepromazine | 3 | 0.1 |
| Doxazosin | 2 | 0.1 |
| Ertugliflozin | 2 | 0.1 |
| Fluvastatin | 2 | 0.1 |
| Levocetirizine | 2 | 0.1 |
| Levodopa | 2 | 0.1 |
| Methotrexate | 2 | 0.1 |
| Rotigotine | 2 | 0.1 |
| Topiramate | 2 | 0.1 |
| Verapamil | 2 | 0.1 |
| Brimonidine | 1 | 0.0 |
| Cannabidiol | 1 | 0.0 |
| Clemastine | 1 | 0.0 |
| Clozapine | 1 | 0.0 |
| Diclofenac | 1 | 0.0 |
| Dimenhydrinate | 1 | 0.0 |
| Glimepiride | 1 | 0.0 |
| Hydroxyzine | 1 | 0.0 |
| Indapamide | 1 | 0.0 |
| Ivermectin | 1 | 0.0 |
| Lisdexamfetamine | 1 | 0.0 |
| Metoclopramide | 1 | 0.0 |
| Naproxen | 1 | 0.0 |
| Rosuvastatin | 1 | 0.0 |
| Tizanidine | 1 | 0.0 |
| **Potential mild alcohol-medication interactions** | **104** | **100** |
| Quetiapine | 89 | 85.6 |
| Abacavir | 10 | 9.6 |
| Doxycycline | 5 | 4.8 |

**SUPPLEMENTARY TABLE 3** Prescribed drugs with potential drug-drug interactions according to mediQ (n = 1622)

| **Drug** | **n** | **%** |
| --- | --- | --- |
| **All drugs involved into potential drug-drug interactions** | **1622** | **100** |
| **Drugs involved in potential severe drug-drug interactions** | **54** | **100** |
| Potassium | 8 | 14.8 |
| Spironolactone | 8 | 14.8 |
| Mirtazapine | 6 | 11.1 |
| Methylphenidate | 5 | 9.3 |
| Sodium bicarbonate | 4 | 7.4 |
| Pipamperone | 3 | 5.6 |
| Chlorprothixene | 2 | 3.7 |
| Mycophenolic acid | 2 | 3.7 |
| Paroxetine | 2 | 3.7 |
| Promethazine | 2 | 3.7 |
| Tacrolimus | 2 | 3.7 |
| Carbamazepine | 1 | 1.9 |
| Citalopram | 1 | 1.9 |
| Clomipramine | 1 | 1.9 |
| Doxepin | 1 | 1.9 |
| Ivabradine | 1 | 1.9 |
| Levomethadone | 1 | 1.9 |
| Lisdexamfetamine | 1 | 1.9 |
| L-thyroxine | 1 | 1.9 |
| Quetiapine | 1 | 1.9 |
| Sertraline | 1 | 1.9 |
| **Drugs involved in potential moderate drug-drug interactions** | **1566** | **100** |
| Pipamperone | 122 | 7.8 |
| Acetylsalycylic acid | 78 | 5.0 |
| Metamizole | 69 | 4.4 |
| Ramipril | 69 | 4.4 |
| Venlafaxine | 67 | 4.3 |
| Mirtazapine | 60 | 3.8 |
| Torasemide | 51 | 3.3 |
| Quetiapine | 44 | 2.8 |
| Doxepin | 43 | 2.7 |
| Spironolactone | 43 | 2.7 |
| Amlodipine | 37 | 2.4 |
| Atorvastatin | 33 | 2.1 |
| Levomethadone | 33 | 2.1 |
| Simvastatin | 31 | 2.0 |
| Carbamazepine | 30 | 1.9 |
| Haloperidol | 29 | 1.9 |
| Candesartan | 26 | 1.7 |
| Chlorprothixene | 24 | 1.5 |
| Buprenorphine | 23 | 1.5 |
| Risperidone | 22 | 1.4 |
| Digitoxin | 18 | 1.1 |
| Hydrochlorothiazide | 18 | 1.1 |
| Allopurinol | 17 | 1.1 |
| Aripiprazole | 17 | 1.1 |
| Bisoprolol | 17 | 1.1 |
| Citalopram | 17 | 1.1 |
| Clonidine | 17 | 1.1 |
| Salbutamol | 17 | 1.1 |
| Sertraline | 17 | 1.1 |
| Fentanyl | 16 | 1.0 |
| Agomelatine | 15 | 1.0 |
| Furosemide | 15 | 1.0 |
| Formoterol | 14 | 0.9 |
| Levomepromazine | 13 | 0.8 |
| Opipramol | 13 | 0.8 |
| Escitalopram | 12 | 0.8 |
| Potassium | 12 | 0.8 |
| Melperone | 12 | 0.8 |
| Bupropion | 11 | 0.7 |
| Olanzapine | 11 | 0.7 |
| Oxazepam | 11 | 0.7 |
| Oxycodone | 11 | 0.7 |
| Fluoxetine | 10 | 0.6 |
| Promethazine | 10 | 0.6 |
| Rivaroxaban | 10 | 0.6 |
| Valproate | 10 | 0.6 |
| Dapagliflozin | 9 | 0.6 |
| Dolutegravir | 9 | 0.6 |
| Donepezil | 9 | 0.6 |
| Duloxetine | 8 | 0.5 |
| Lorazepam | 8 | 0.5 |
| Apixaban | 7 | 0.4 |
| Digoxin | 7 | 0.4 |
| L-thyroxine | 7 | 0.4 |
| Prednisolone | 7 | 0.4 |
| Trimipramine | 7 | 0.4 |
| Ibuprofen | 6 | 0.4 |
| Lithium | 6 | 0.4 |
| Tacrolimus | 6 | 0.4 |
| Tramadol | 6 | 0.4 |
| Valsartan | 6 | 0.4 |
| Amisulpride | 5 | 0.3 |
| Fluconazole | 5 | 0.3 |
| Methylphenidate | 5 | 0.3 |
| Phenprocoumon | 5 | 0.3 |
| Sacubitril | 5 | 0.3 |
| Trazodone | 5 | 0.3 |
| Vitamin D | 5 | 0.3 |
| Iron | 4 | 0.3 |
| Enalapril | 4 | 0.3 |
| Eplerenone | 4 | 0.3 |
| Flupentixol | 4 | 0.3 |
| Insulin | 4 | 0.3 |
| Levetiracetam | 4 | 0.3 |
| Metoprolol | 4 | 0.3 |
| Pantoprazole | 4 | 0.3 |
| Tenofovir | 4 | 0.3 |
| Xipamide | 4 | 0.3 |
| Clomipramine | 3 | 0.2 |
| Dabigatran | 3 | 0.2 |
| Milnacipran | 3 | 0.2 |
| Naltrexone | 3 | 0.2 |
| Paroxetine | 3 | 0.2 |
| Prasugrel | 3 | 0.2 |
| Salmeterol | 3 | 0.2 |
| Tizanidine | 3 | 0.2 |
| Amitriptyline | 2 | 0.1 |
| Atomoxetine | 2 | 0.1 |
| Chlorthalidone | 2 | 0.1 |
| Ciprofloxacin | 2 | 0.1 |
| Cobicistat | 2 | 0.1 |
| Darunavir | 2 | 0.1 |
| Diazepam | 2 | 0.1 |
| Enoxaparin | 2 | 0.1 |
| Esomeprazole | 2 | 0.1 |
| Fluvoxamine | 2 | 0.1 |
| Indacaterol | 2 | 0.1 |
| Levofloxacin | 2 | 0.1 |
| Mycophenolic acid | 2 | 0.1 |
| Sodium bicarbonate | 2 | 0.1 |
| Pregabalin | 2 | 0.1 |
| Verapamil | 2 | 0.1 |
| Adalimumab | 1 | 0.1 |
| Aluminum hydroxide | 1 | 0.1 |
| Budesonide | 1 | 0.1 |
| Calcium | 1 | 0.1 |
| Cannabidiol | 1 | 0.1 |
| Carvedilol | 1 | 0.1 |
| Clarithromycin | 1 | 0.1 |
| Clemastine | 1 | 0.1 |
| Clopidogrel | 1 | 0.1 |
| Cobalamin | 1 | 0.1 |
| Cotrimoxazole | 1 | 0.1 |
| Dimenhydrinate | 1 | 0.1 |
| Dulaglutide | 1 | 0.1 |
| Empagliflozin | 1 | 0.1 |
| Folic acid | 1 | 0.1 |
| Hydromorphone | 1 | 0.1 |
| Hydroxyzine | 1 | 0.1 |
| Indapamide | 1 | 0.1 |
| Lacosamide | 1 | 0.1 |
| Lamotrigine | 1 | 0.1 |
| Levodopa | 1 | 0.1 |
| Metformin | 1 | 0.1 |
| Morphine | 1 | 0.1 |
| Nebivolol | 1 | 0.1 |
| Ofloxacin | 1 | 0.1 |
| Olodaterol | 1 | 0.1 |
| Piracetam | 1 | 0.1 |
| Pyridoxine | 1 | 0.1 |
| Ranolazine | 1 | 0.1 |
| Sitagliptin | 1 | 0.1 |
| Tilidine | 1 | 0.1 |
| Vilanterol | 1 | 0.1 |
| **Not beneficial because same mechanism of action** | **2** | **100** |
| Furosemide | 1 | 50.0 |
| Torasemide | 1 | 50.0 |
